# Green Nail in a Patient With Psoriasis Receiving Deucravacitinib

**DOI:** 10.7759/cureus.75906

**Published:** 2024-12-17

**Authors:** Yoshihito Mima, Tsutomu Ohtsuka, Yuta Norimatsu

**Affiliations:** 1 Department of Dermatology, Tokyo Metropolitan Police Hospital, Tokyo, JPN; 2 Department of Dermatology, International University of Health and Welfare Hospital, Nasushiobara, JPN; 3 Department of Dermatology, International University of Health and Welfare Narita Hospital, Narita, JPN

**Keywords:** deucravacitinib, green nail, interleukin-10, pseudomonas aeruginosa, tyrosine kinase 2

## Abstract

Green nail (GN) is typically caused by *Pseudomonas aeruginosa* and commonly occurs in patients with nail damage, nail psoriasis, or frequent exposure to moist environments. Deucravacitinib is an oral tyrosine kinase 2 (Tyk2) inhibitor effective for psoriasis treatment. Herein, we report a case of GN in a 72-year-old man following four months of treatment with deucravacitinib. Given the absence of pre-existing signs indicative of nail psoriasis prior to the initiation of deucravacitinib, deucravacitinib might have contributed to the development of GN. To the best of our knowledge, no previous cases of GN associated with deucravacitinib or studies discussing a potential link between the two have been reported. Interleukin (IL)-10 plays a crucial role in regulating T-cell immune responses specific to *P. aeruginosa*. Tyk2 mediates IL-10 signaling and promotes IL-10 activation. In this case, the inhibition of IL-10 function via Tyk2 suppression due to deucravacitinib might contribute to the development of GN.

## Introduction

Persistent greenish pigmentation of the nail plate was originally described in 1944 by Goldman and Fox, who linked it to nail plate involvement by *Pseudomonas aeruginosa *(*P. aeruginosa*) [1]. In the field of dermatology today, a nail with a green appearance is referred to as green nail (GN) [2]. GN has been described as a triad of green discoloration of the nail plate, proximal paronychia, and distal onycholysis [3]. GN is most commonly caused by *P. aeruginosa* [4].

*P. aeruginosa* is a gram-negative, aerobic coccobacillus that is ubiquitous in nature [5]. It produces the blue-green pigments pyocyanin and pyoverdine that contribute to the characteristic green discoloration of the nail and sometimes detectable green fluorescence due to the fluorescent siderophore pyoverdine [6]. Internally, *P. aeruginosa* is an opportunistic pathogen responsible for a wide variety of diseases, including pneumonia, otitis externa, urinary tract infections, osteomyelitis, and sepsis [5]. Since the dermis is dry, this pathogen cannot survive on the skin, making cutaneous infections rare. It is not part of the normal flora of healthy human skin [5]. However, with exposure to moist environments, *P. aeruginosa* can cause cutaneous infections that may spread. The increased risk of serious and life-threatening *P. aeruginosa* infections, such as necrotizing fasciitis and ecthyma gangrenosum, is particularly concerning in immunocompromised patients [5]. Therefore, individuals with compromised immune function, frequent exposure to water, soap, or detergents, or nail damage due to trauma, nail psoriasis, or onychomycosis are particularly susceptible to GN [2-5]. Topical treatment with antibacterial agents, such as nadifloxacin, is often effective in managing GN caused by *P. aeruginosa* [2,3]. Complete resolution of GN is typically achieved within 4 to 6 months [2].

Psoriasis is a chronic, immune-mediated, inflammatory skin disease that significantly impacts patients’ quality of life [7,8]. It typically presents as sharply demarcated erythematous plaques covered by silvery-white lamellar scales, which may develop in regions such as the scalp, gluteal fold, elbows, and knees [9]. Psoriasis has a complex pathogenesis associated with several systemic comorbidities, such as psoriatic arthritis, cardiovascular disease, metabolic syndrome, obesity, diabetes, hypertension, autoimmune disorders, malignancies, inflammatory bowel disease, nonalcoholic fatty liver disease, and depression [10].

In psoriasis vulgaris, T helper (Th)1 and Th17 cells play critical roles in disease pathogenesis. Interleukin (IL)-12 is essential for the differentiation and proliferation of Th1 cells, which produce interferon-γ and tumor necrosis factor-α, while IL-23 is central to the survival and expansion of Th17 cells [11]. Consequently, IL-12 and IL-23 are considered key pathogenic mediators in psoriasis vulgaris [11]. Activated Th1 and Th17 cells release cytokines such as IL-17A, IL-17F, IL-22, and tumor necrosis factor-α, leading to excessive keratinocyte proliferation and inflammation in both the epidermis and dermis [11-13].

In addition to injectable therapies targeting IL-17 and IL-23, which drive keratinocyte hyperproliferation and inflammation through Th17 cell activation, a novel oral treatment, deucravacitinib, has emerged for psoriasis vulgaris [14,15]. Deucravacitinib is a selective oral tyrosine kinase 2 (TYK2) inhibitor that is administered orally [15]. TYK2, a member of the JAK family, mediates signaling pathways for IL-12, IL-23, and type I interferons [16]. By selectively inhibiting TYK2, deucravacitinib disrupts these signaling pathways, mitigating keratinocyte hyperproliferation and excessive inflammation, making it an effective treatment for psoriasis vulgaris [15,16].

The clinical efficacy of deucravacitinib has been demonstrated in two large phase III trials comparing it to placebo and apremilast in patients with psoriasis vulgaris [15]. Common side effects of deucravacitinib include nasopharyngitis, upper respiratory tract infections, headache, diarrhea, and nausea. Unlike other Janus kinase inhibitors, deucravacitinib has a favorable safety profile, with a low incidence of severe infections, thromboembolic events, or significant laboratory abnormalities [15]. To date, there have been no reported cases of GN development in patients receiving deucravacitinib for psoriasis vulgaris.

Herein, we report a case of GN that developed after the initiation of deucravacitinib therapy for psoriasis vulgaris in the absence of frequent exposure to moist environments or nail trauma.

## Case presentation

**Figure 1:**
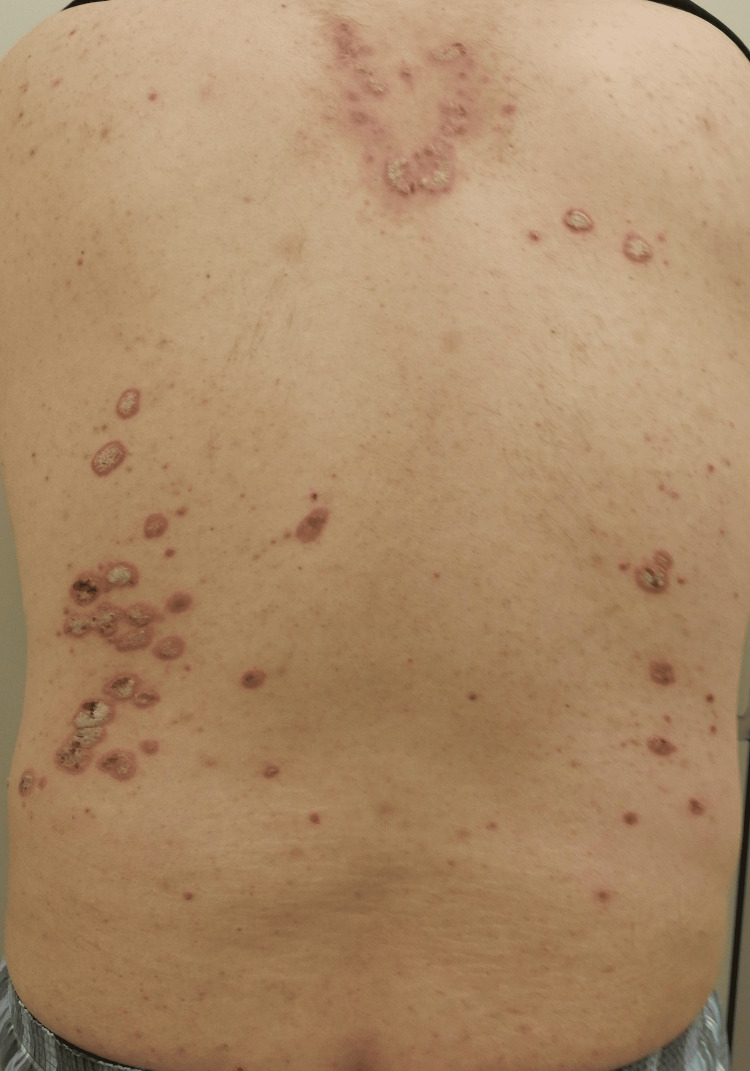
Edematous erythema with scales on the trunk accompanied by partial pigmentation

A 72-year-old man with a 10-year history of psoriasis was administered topical corticosteroids and calcipotriol. However, because his psoriasis worsened with topical treatment alone, he was referred to our dermatology department. In addition to psoriasis, he had a history of hypertension, but he had no history of any other immunological diseases or relevant conditions. Physical examination revealed scattered erythematous plaques with scaling on his limbs and trunk (Figure 1).

**Figure 2:**
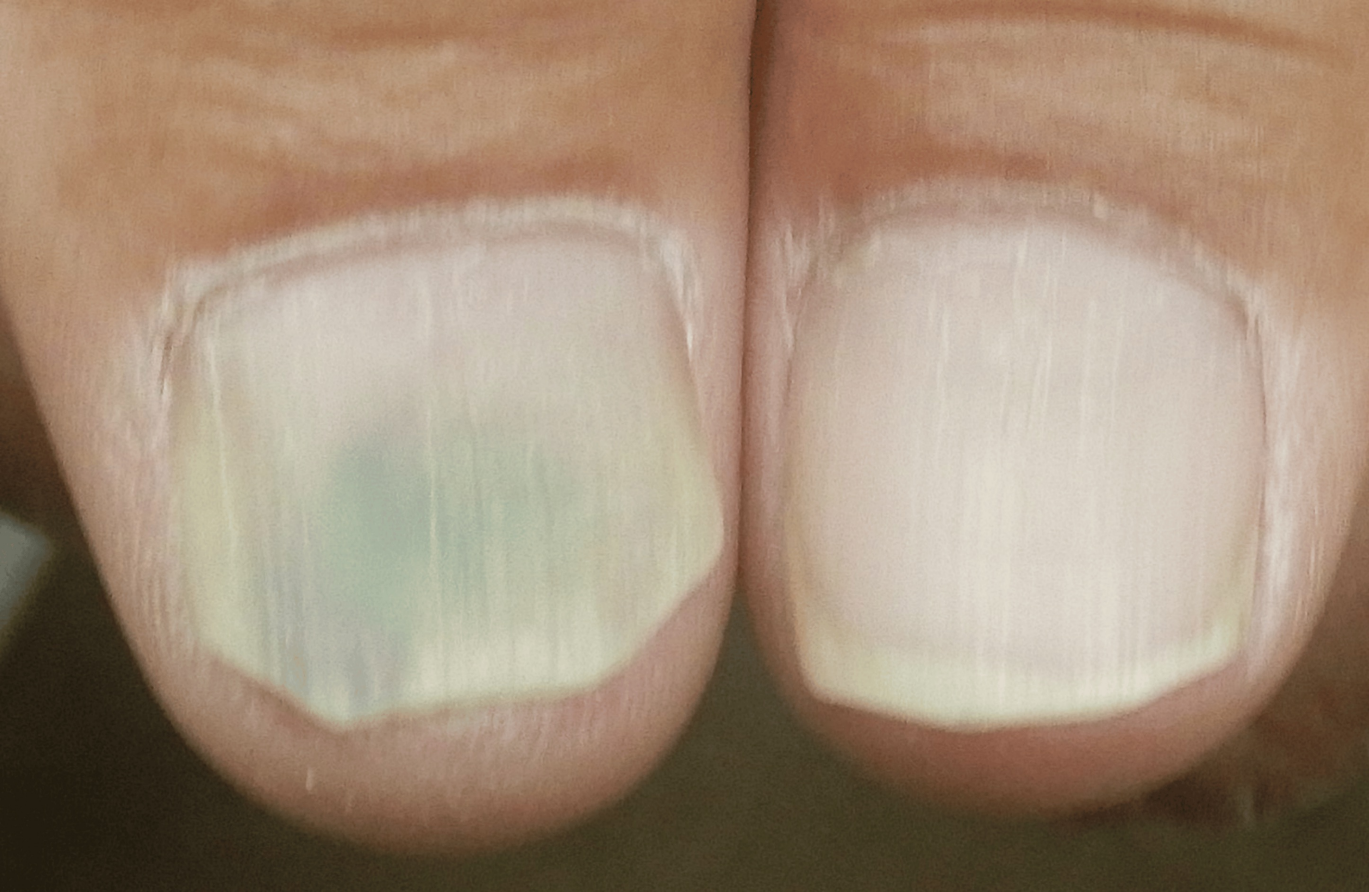
Right thumb nail with green-colored staining and left thumb nail without nail psoriasis

His psoriasis area and severity index (PASI) score was 18.4. No systemic symptoms, including arthritis or gastrointestinal disorders, were observed. Given the severity of his condition, we considered intensifying treatment with biological agents or oral Tyk2 inhibitors. However, the patient preferred oral medication. Therefore, after confirming that his blood test, urine test, and chest radiography findings were normal, deucravacitinib treatment was initiated. The topical medication was also switched to a combination of calcipotriol hydrate and betamethasone dipropionate. After four months of deucravacitinib treatment, the patient’s PASI score improved to 4.2. However, GN was observed at the four-month follow-up visit (Figure 2).

**Figure 3:**
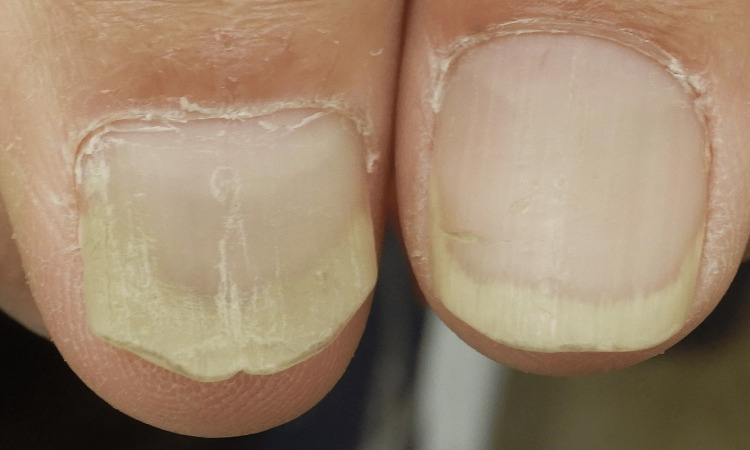
Green nail showed complete regression following the application of 1% nadifloxacin ointment for four months

The patient had no history of nail trauma or frequent exposure to water. Furthermore, nail psoriasis was not observed before deucravacitinib was initiated. Therefore, we considered the possibility that the occurrence of GN was associated with deucracitinib administration. Bacterial cultures of the nail did not detect any bacteria, and repeated fungal tests were negative. Considering that bacterial cultures for GN often yield false negatives and that we suspected *P. aeruginosa* infection, we initiated topical treatment with 1% nadifloxacin [17]. The GN resolved showed significant improvement two months after starting the antibacterial ointment and achieved complete resolution at four months (Figure 3).

## Discussion

Deucravacitinib is an oral medication for psoriasis vulgaris that selectively inhibits Tyk2 [15]. The reported adverse effects of deucravacitinib include nasopharyngitis, upper respiratory tract infections, headache, diarrhea, nausea, severe infections, thromboembolic events, and significant laboratory abnormalities [15]. However, these side effects are rare, making it a highly safe treatment, with very few patients requiring discontinuation due to adverse effects [15]. To the best of our knowledge, no cases of GN development in patients with psoriasis vulgaris following deucravacitinib initiation have previously been reported.

IL-10 plays a critical role in controlling T-cell immune responses specific to *P. aeruginosa* [18]. Tyk2 mediates IL-10 signaling and promotes interferon-gamma-dependent IL-10 activation [19]. Therefore, inhibition of Tyk2 signaling by deucravacitinib may impair the IL-10 signaling pathway and IL-10 activation, potentially weakening the T-cell immune response against *P. aeruginosa* [18,19]. This may have led to bacterial proliferation and GN development in our case [18,19]. To date, no studies have discussed the association between JAK inhibitors and green nail syndrome. However, since both Tyk2 and JAK1 are involved in IL-10 signaling and promote IL-10 activation, JAK1 inhibitors such as upadacitinib may, like Tyk2 inhibitors, impair the IL-10 signaling pathway and IL-10 activation, potentially weakening the T-cell immune response against *P. aeruginosa* and having the possibility of GN development [20].

In the present case, GN developed despite the absence of risk factors such as nail psoriasis, nail trauma, or frequent exposure to moist environments. Therefore, we believe that the inhibition of IL-10 function caused by deucravacitinib led to a weakened T-cell immune response against *P. aeruginosa*, significantly contributing to the development of GN. As an opportunistic pathogen, *P. aeruginosa* can cause severe, life-threatening infections. GN caused by *P. aeruginosa* poses a risk of nosocomial infections, as it may spread through contact and infect immunocompromised elderly individuals. Therefore, caution is necessary, and early detection and treatment are crucial [1-5].

To the best of our knowledge, no studies have reported a correlation between deucravacitinib therapy and the development of GN. Since Tyk2 functions as a downstream signaling molecule for many cytokines [15,18,19], broad suppression of these cytokines, unlike the targeted inhibition observed with biological agents, may result in unexpected side effects. As deucravacitinib is a relatively new drug [15], unforeseen side effects, such as those observed in this case, may be observed in the future. Therefore, further case reports and research on the side effects of deucravacitinib are essential.

## Conclusions

In the present case, we discussed the possibility that inhibition of IL-10 function via Tyk2 suppression by deucravacitinib contributed to the development of GN. Tyk2 functions as a downstream signaling molecule for many cytokines, so broad suppression of cytokines by deucravacitinib may result in unexpected side effects, as observed in this case. GN caused by *P. aeruginosa* poses a risk of nosocomial infections through contact transmission and may lead to severe, life-threatening infections, particularly in immunocompromised elderly individuals. Therefore, early detection and treatment of GN are crucial. As deucravacitinib is a relatively new drug, unexpected side effects, such as GN, may continue to be reported. Thus, further case reports and research on the side effects of deucravacitinib are essential.
